# Reduction of intra-abdominal pain through transcranial direct current stimulation: A systematic review

**DOI:** 10.1097/MD.0000000000017017

**Published:** 2019-09-27

**Authors:** Kian-Elias Bayer, Lars Neeb, Arian Bayer, Jakob Johann Wiese, Britta Siegmund, Magdalena Sarah Prüß

**Affiliations:** a Medizinische Klinik m. S. Gastroenterologie, Infektiologie und Rheumatologie, Charité -Universitätsmedizin Berlin; b Department of Neurology, Berlin Charité - Universitätsmedizin Berlin, corporate member of Freie Universität Berlin, Humboldt-Universität zu Berlin, and Berlin Institute of Health; c Berlin Institute of Health (BIH), Berlin, Germany.

**Keywords:** abdominal pain, intra-abdominal pain, transcranial direct current stimulation, visceral pain

## Abstract

**Background:**

Transcranial direct current stimulation (tDCS) is a non-invasive brain stimulation technique to modulate cortical excitability and to induce neuronal plasticity. With a wide range of applications in neurological and psychiatric disorders, the efficiency of tDCS is also studied in the treatment of various pain conditions. Treatment with tDCS might accordingly provide pain relief for patients with acute or chronic pain and thus lead to an increase in quality of life. Moreover, applied as an adjunct therapy, tDCS can reduce help to reduce pain medication intake and accompanying adverse events. To this end, this review examines studies evaluating the efficacy of tDCS in pain relief in patients with intra-abdominal pain.

**Methods:**

A structured search of the PubMed medical database was carried out to identify possibly relevant studies. Studies were compared in terms of treatment characteristics, general conditions, and results. Jadad scale was applied for quality analyses.

**Results:**

Out of 289 articles that were found initially, 6 studies were identified that met eligibility criteria. Five out of 6 studies reported significant effects for pain reduction in different types of intra-abdominal pain.

**Conclusions:**

Results indicate that tDCS might be able to reduce intra-abdominal pain. However, more randomized-controlled trials with larger sample size are necessary to define clinically relevant effects as well as treatment characteristics such as duration of stimulation.

## 1 Introduction

### 1.1 Transcranial direct current stimulation

Transcranial direct current stimulation (tDCS) is an increasingly tested technique for non-invasive electrostimulation of the brain with the effect of inducing a modulation in cortical excitability.^[1]^ This neuromodulatory technique uses direct currents with a low amplitude delivered through the scalp via surface electrodes. The effects of a tDCS session vary from immediate to long-term effects depending largely on the type of stimulation selected as well as current density/intensity and duration of stimulation and last beyond the duration of stimulation.^[2–4]^ Explanation attempts for the immediate effects of tDCS indicate stimulation-dependent changes of the resting membrane potential of neurons—in which two forms of stimulation are being distinguished. The stimulation of a brain region through a cathodal electrode decreases the resting membrane potential, resulting in alterations of spontaneous neural firing and discharge rates, finally causing a hyperpolarization of neurons and a decrease in cortical excitability.^[3,5]^ The anodal form of stimulation on the contrary, results in the opposite by increasing the resting membrane potential, thus eventually leading to depolarization and to an increase of excitation in cortical circuits.^[4–6]^ Possible explanations for the prolonged effects and changes in cortical excitability are that tDCS induces changes in cortical synaptic transmission, similar to the long-term potentiation and long-term-depression and an enhancement of protein synthesis resulting in alterations of synaptic plasticity.^[7–9]^ So far, tDCS was studied in a variety of clinical conditions and applications such as treatment of major depression,^[10]^ stroke rehabilitation,^[11]^ and modulation of perception of acute and chronic pain.^[12]^ This rapidly growing field of research is requiring investigation of the effectiveness of tDCS.

This systematic review aims to summarize current literature and studies on tDCS for intra-abdominal pain.

## 2 Material and methods

As this is a review, ethical approval was not required.

### 2.1 Search strategy

The medical database “Pubmed” was searched for medical studies. No time limit has been set regarding the year of publication of the results. The search was conducted on January 2, 2019. The following key words were used: (transcranial direct current stimulation OR transcranial current stimulation OR tDCS) and (abdominal pain OR visceral pain OR pelvic pain OR pancreatic pain OR surgery pain OR chronic pain).

### 2.2 Selection criteria

The following inclusion criteria were applied: articles written in English or German; and placebo-controlled randomized controlled trials (RCT) were included. Exclusion criteria were set as following: animal studies; review articles, case report series, articles that focused only on the effects of other brain stimulation techniques such as transcranial alternating current stimulation.

### 2.3 Data extraction

Data extraction was performed based on elaborated spreadsheets. The included studies were compared and screened considering the following data:

1. Treatment characteristics including electrode positioning of anode and cathode, size of electrodes, intensity of electric current, current densities, duration of each stimulation-session and duration of intervention (amount of repetitions/days of stimulation).
2. Clinical characteristics and quality assessment, such as sample size, distribution of sex, and age-average.
3. Methodological procedure to assess potential pain reduction within the study, experimental results, and conclusions of the authors on tDCS and its effects on pain perception in patients with intra-abdominal pain.

### 2.4 Quality assessment

The methodological quality of RCT's was assessed based on the Jadad scale.^[13]^ This is a scale with 5 dichotomous questions, where 1 question corresponds to 1 point. Studies with a score below 3 points indicate a poor quality of the study. In order to evaluate the RCT's on the basis of the Jadad scale, the following aspects were extracted from each study: randomization, blinding, drop-outs.

## 3 Results

### 3.1 Search results

**Figure 1:**
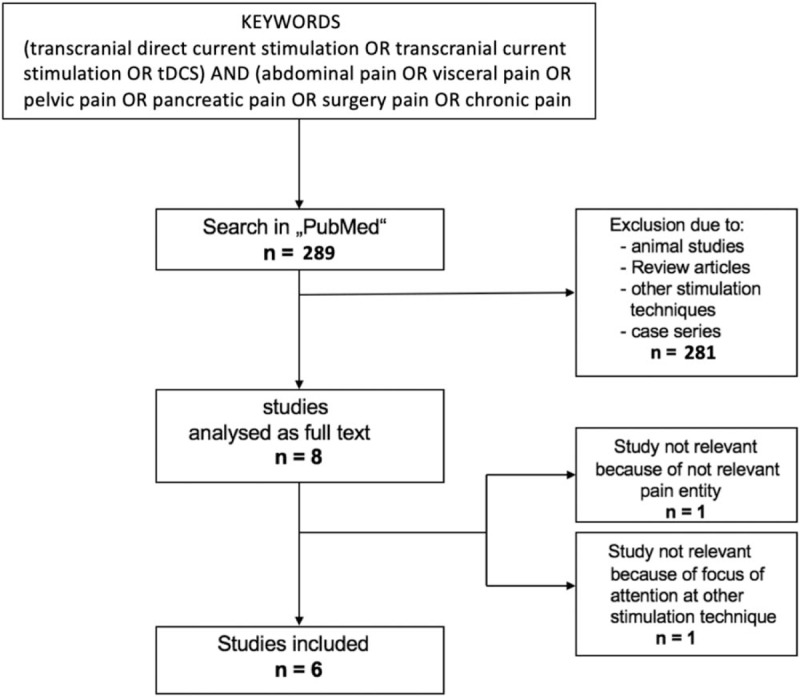
Flowchart of literature search. Progress of the literature search. The search was conducted on “Pubmed” with the described keywords. One hundred ninety three entries were found initially. Due to exclusion criteria, 8 studies remained after reading the title and abstract. Two further studies were excluded after reading the full text—resulting in a total of 6 studies which were included in the review.

The selection process in the search for potential studies is shown in Fig. 1. Including the keywords listed above, 289 articles were found. After excluding studies according to the selection criteria and due to the title and abstract 8 articles remained. The exclusion of these studies was primarily due to the use of tDCS for pain relief in chronic pain conditions other than intra-abdominal pain (such as chronic pain caused by fibromyalgia) or due to the circumstance that the studies were conducted in animals. One study had to be excluded as it was a case report (1 patient with endometriosis).^[14]^ After reviewing 8 full text articles 2 other studies were excluded. The first study had to be excluded due to the incident that tDCS was only tested in combination with other stimulation procedures, thus making it impossible to objectively assess the sole effects of tDCS on abdominal pain reduction.^[15]^ Although the second excluded study tested tDCS for pain reduction in patients with hepatitis C, reading the full text revealed that the pain did not belong to abdominal pain but headache.^[16]^ Eventually, a total of 6 studies were identified which complied with the overall criteria.^[17–22]^

### 3.2 Study characteristics and quality criteria

The main treatment and study characteristics as well as the quality criteria of the 6 RCT's^[17–22]^ are summarized in Tables 1 and 2.

**Table 1:**
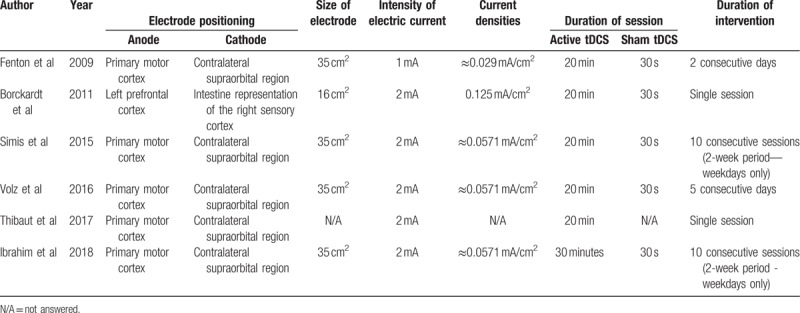
Treatment characteristics such as electrode positioning, duration of session, and duration of intervention.

**Table 2:**
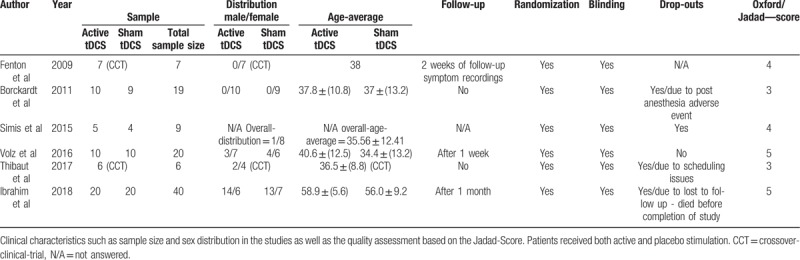
Clinical characteristics and quality assessment.

Both the duration of the actual interventions within the studies as well as the performed follow-up examinations were included in the evaluation. The duration of the intervention varied between a single session and 10 stimulation-sessions.

Aside from one exception all studies applied a stimulation form in which the anode was placed above the motor cortex and the cathode was placed over the contralateral supraorbital region.^[17,19–22]^ One study deviated from this pattern and placed the anode above the prefrontal cortex, while the cathode was attached to the intestine representation of the right sensory cortex.^[18]^ Further differences in the study characteristics were found regarding the size of the electrodes used and the intensity of electric current. As a consequence, there was also a bandwidth of different current densities, which we have additionally calculated as mA/cm^2^. In 4 studies the applied electrodes were both in the size of 35 cm^2^.^[17,19,20,22]^ One study used electrodes both with a size of 16 cm^2^^[18]^ and in another study the size of electrodes was not specified.^[21]^ The intensity of electric current differed between 1^[17]^ and 2 mA.^[18–22]^ In one study the current density was approximately 0.029 mA/cm^2^.^[17]^ In another study, the current density could not be calculated due to the lack of information about the electrode size.^[21]^ In 3 studies the current density was at approximately 0.0571 mA/cm^2^^[19,20,22]^ and in another study the current density reached 0.125 mA/cm^2^.^[18]^ Differences were also found regarding the duration of individual stimulation-sessions, which usually equaled 20 minutes.^[17–21]^ Only one study used a 30 minutes stimulation on 10 consecutive days.^[22]^

In terms of quality characteristics, the studies displayed a relatively broad heterogeneity. Two studies were rated with a 3 on the Jadad scale.^[18,21]^ Two other studies were rated with a 4^[17,19]^ and another 2 studies received 5 out of 5 points.^[20,22]^ Double-blinding within the studies was not always adequately described—3 studies did not clearly show to what extent the assessors and researchers conducting the tDCS treatment were blinded.^[18–20]^

### 3.3 Study results

In the 6 identified studies, a total of 101 patients were treated with either active or sham-tDCS. The main outcomes and results as well as the conclusions of the authors have been summarized in Table 3 .

**Table 3:**
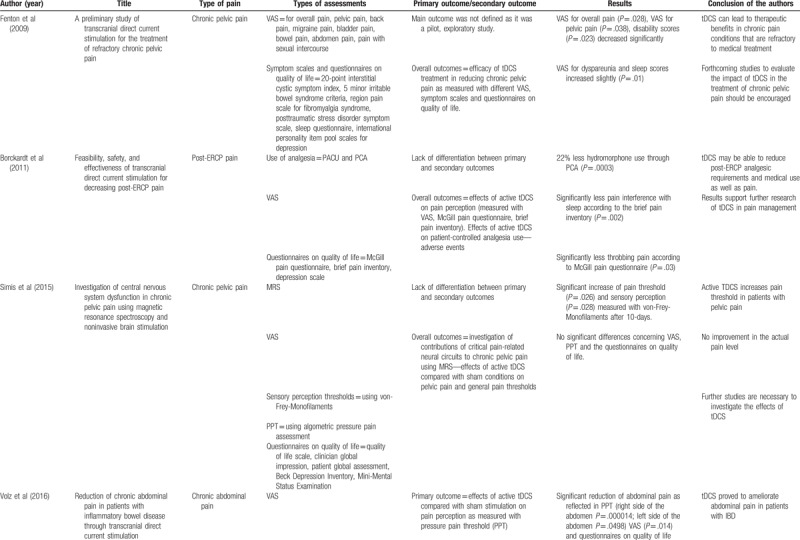
Assessments used in the studies as well as results and conclusion of the authors.

**Table 3 (Continued):**
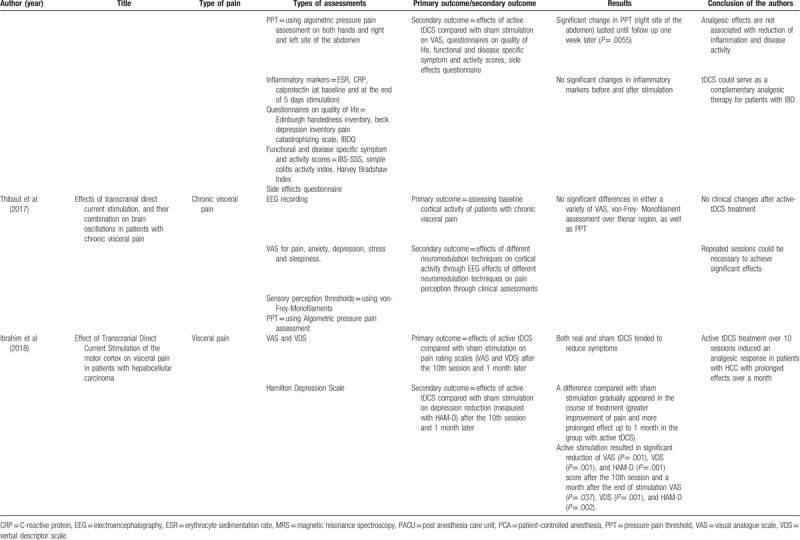
Assessments used in the studies as well as results and conclusion of the authors.

All studies evaluated the intensity of pain with visual analogue scales (VAS). Five Studies used questionnaires on quality of life and a variety of symptom scores.^[17–20,22]^ Three studies made use of an algometric pressure pain assessment in order to evaluate the pressure pain threshold (PPT).^[19–21]^ Two studies measured the sensory perception threshold using von-Frey-Monofilaments.^[19,21]^

A total of 5 studies reported that tDCS relieved intra-abdominal pain sensations measured with some of the used assessments,^[17–20,22]^ whereas 1 study did not demonstrate any difference between sham and active stimulation regarding pain reduction.^[21]^ Fenton et al^[17]^ investigated the efficacy of tDCS in the therapy of patients with chronic pelvic pain in a cross-over study. Active tDCS treatment resulted in a significant decrease of different VAS. Another 5 out of 6 studies reported side effects, which were limited to mild tingling, itching, and burning sensations as well as skin redness, scalp, or neck pain.^[17–20,22]^

Borckardt et al^[18]^ enrolled patients who were expected to receive an endoscopic retrograde cholangiopancreaticography (ERCP) and assessed the efficacy and safety of tDCS on post-ERCP pain and analgesia use. Patients who received active stimulation used 22% less hydromorphone compared with the sham group and reported significantly less throbbing abdominal pain.

Simis et al^[19]^ also studied the effects of tDCS in pain reduction in patients with chronic pelvic pain. In addition, the study aimed to assess the contributions of biochemical metabolites in pain related neural circuits to pelvic pain. Roles of 4 different brain areas were evaluated measuring various metabolites using Magnet-Resonance-Spectroscopy. Pain threshold and sensory perception as measured with von-Frey-Monofilaments increased significantly after active tDCS treatment, whereas VAS, PPT (using algometric pressure pain assessment), and questionnaires on quality of life did not differ.

In our own study (Volz et al^[20]^), we aimed to investigate the effects of tDCS in patients with chronic abdominal pain due to inflammatory bowel disease. Primary outcome was to determine the effects of tDCS on PPT. Different inflammation markers were measured to ensure that pain reduction was not simply induced by a decrease in inflammation. Active tDCS led to significant pain reduction as reflected in PPT, VAS, and questionnaires on quality of life. Pain reduction remained significantly decreased in the right side of the abdomen after 1 week at the follow-up.

Thibaut et al^[21]^ included participants with chronic pelvic pain to receive interventions with different neuromodulation techniques in order to compare the effects of transcranial pulsed current stimulation (tPCS) and tDCS combined, tPCS alone, tDCS alone, and sham condition on pain assessment and cortical activity (measured with EEG). No significant differences between active and sham stimulations were found. In the study of Ibrahim et al^[22]^ patients with hepatocellular carcinoma associated with visceral pain underwent 10 sessions of tDCS. Although both sham and active tDCS reduced abdominal pain, a significant difference in the active group was shown successively throughout the course of treatment in comparison to the control group. The significant pain reduction lasted to the follow-up a month after the last stimulation.

## 4 Discussion

Based on neurobiological hypotheses and examinations about the long-term effects of tDCS on modulation of corticospinal circuits,^[7–9]^ the results of the investigated studies may lead to the conclusion that patients with intra-abdominal pain benefit from treatment with tDCS, since tDCS therapy results in a significant reduction of pain. It appears that treatment with tDCS increases both the perception threshold, and the pain threshold of patients with intra-abdominal pain of different entities. Thus, nearly all clinical studies demonstrated significant effects in pain reduction as measured with VAS, von-Frey-Monofilaments, and pain pressure algometry as well as general improvements in quality of life as measured with different questionnaires. Furthermore, the effects of tDCS seem to be cumulative, meaning that effects on pain reduction last longer the more stimulation sessions are conducted. However, it is necessary to consider that a potentially improved quality of life through tDCS does not have to be attributed to pain reduction alone. It might be a combination of both analgesic and antidepressant influences due to an antidepressant effect of tDCS.^[23]^ As far as reported tDCS appears to be a safe procedure without severe side effects and seems to be limited to tingling, itching, mild burning sensations as well as skin redness and mild headache or neck pain during the stimulation.

There are limitations of this systematic review to be named. To some extent these limitations result from the circumstance that only one medical database was searched for potentially suitable studies. Consequently, it cannot be ruled out that a search in other databases could have led to the inclusion of further studies. Moreover, it cannot be precluded that studies were mistakenly excluded in the selection process. A further limitation of this review is the small number of 6 included studies, each of which with a comparatively small sample size. Limitations of the included studies are mainly due to the heterogeneity, reflected in the variety of different treatment characteristics. For instance, the duration of intervention in the studies differed substantially ranging between a single session and 10 consecutive stimulation sessions. Considering that the effects of tDCS are presumably based on mechanisms similar to those of long-term potentiation, the question arises to what extent the effects can be achieved through a single session of stimulation. Furthermore, the size of electrodes used for stimulation, as well as the intensity of electric current varied considerably between studies. These variable treatment characteristics have an overall effect on the current densities for stimulation. The current densities consequently ranged from ≈0.029 to 0.125 mA/cm^2^, making the comparability difficult at some points. Another consideration which might be a general problem in the application of tDCS and could also complicate the comparability of studies are interindividual morphological variabilities. Since the cranial bones of the participants are varying in size and thickness, the electrical resistance differs with each stimulation. Hair density should also have an influential impact. As both hair and bone tissue are insufficient electrical conductors, they should have an influence on the actually achieved intracranial current density. In conclusion, although it has not been completely clarified how tDCS works on a neuromodulatory basis, the results of the studies indicate that tDCS may be able to reduce intra-abdominal pain and therefore improve patients’ quality of life. Moreover, an adjunct tDCS treatment could lead to a reduction of pain medication intake and secondarily to a reduction of the accompanying adverse events of pain medication (such as bowel paralysis due to opioids). To what extent the immediate and prolonged effects of tDCS depend on parameters such as duration of stimulation sessions as well as the duration of the complete intervention needs to be clarified in further studies. In addition, greater value should be placed on sufficient blinding. This could lead up to triple-blinded-trials in which patients, as well as evaluators and tDCS-operators are all unaware of the treatment the participant receives. Moreover, future RCT's should include a larger sample size and should be designed as phase-III-studies in order to determine clinical relevance of analgesic effects of tDCS as one of the key problems within the research area of tDCS is the lack of large-scaled, multicentered studies. Thus, the potential efficacy of tDCS has yet to be fully assessed. However the data so far present strong hints that tDCS has a beneficial effect on chronic pain syndromes.

## Author contributions

**Conceptualization:** Kian-Elias Bayer, Lars Neeb, Arian Bayer, Britta Siegmund, Magdalena Sarah Prüß.

**Data curation:** Kian-Elias Bayer, Magdalena Sarah Prüß.

**Investigation:** Kian-Elias Bayer, Arian Bayer, Magdalena Sarah Prüß.

**Project administration:** Kian-Elias Bayer, Britta Siegmund, Magdalena Sarah Prüß.

**Supervision:** Britta Siegmund, Magdalena Sarah Prüß.

**Writing – original draft:** Kian-Elias Bayer.

**Writing – review & editing:** Kian-Elias Bayer, Lars Neeb, Arian Bayer, Jakob Johann Wiese, Britta Siegmund, Magdalena Sarah Prüß.
